# An approach to describing and analysing bulk biological annotation quality: a case study using UniProtKB

**DOI:** 10.1093/bioinformatics/bts372

**Published:** 2012-09-03

**Authors:** Michael J. Bell, Colin S. Gillespie, Daniel Swan, Phillip Lord

**Affiliations:** ^1^School of Computing Science; ^2^School of Mathematics & Statistics; ^3^Bioinformatics Support Unit, ICAMB, Medical School, Newcastle University, Newcastle-Upon-Tyne, NE1 7RU, UK

## Abstract

**Motivation:** Annotations are a key feature of many biological databases, used to convey our knowledge of a sequence to the reader. Ideally, annotations are curated manually, however manual curation is costly, time consuming and requires expert knowledge and training. Given these issues and the exponential increase of data, many databases implement automated annotation pipelines in an attempt to avoid un-annotated entries. Both manual and automated annotations vary in quality between databases and annotators, making assessment of annotation reliability problematic for users. The community lacks a generic measure for determining annotation quality and correctness, which we look at addressing within this article. Specifically we investigate word reuse within bulk textual annotations and relate this to Zipf's Principle of Least Effort. We use the UniProt Knowledgebase (UniProtKB) as a case study to demonstrate this approach since it allows us to compare annotation change, both over time and between automated and manually curated annotations.

**Results:** By applying power-law distributions to word reuse in annotation, we show clear trends in UniProtKB over time, which are consistent with existing studies of quality on free text English. Further, we show a clear distinction between manual and automated analysis and investigate cohorts of protein records as they mature. These results suggest that this approach holds distinct promise as a mechanism for judging annotation quality.

**Availability:** Source code is available at the authors website: http://homepages.cs.ncl.ac.uk/m.j.bell1/annotation.

**Contact:** phillip.lord@newcastle.ac.uk

## 1 INTRODUCTION

A key descriptive feature of biological data is its *annotation*: a textual representation of the biology associated with the data. Biologists use these annotations to understand and contextualize data in biological sequence databases. Annotations play an essential role in describing and developing the users' knowledge of a given sequence and can form the foundation for further research (Jones *et al.*, 2007). Some annotation is structured, for example, using an ontology (Stevens and Lord, 2009) or keyword list. However, free text annotation often contains the richest biological knowledge (Camon *et al.*, 2005), but while free text is appropriate for human comprehension it is difficult to interpret computationally.

The current ‘gold standard’ for annotation is a set of reviewed and manually curated entries (Curwen *et al.*, 2004). However, manually-curated annotation is labour-intensive, time consuming and costly. To cope with the amount of data, which is typically increasing exponentially, many resources and projects generate annotations computationally (Boeckmann *et al.*, 2003). Automated annotations are more prone to errors than their manual counterparts (Gilks *et al.*, 2002), with several studies suggesting high levels of misannotation in automated annotation (Andorf *et al.*, 2007; Jones *et al.*, 2007; Schnoes *et al.*, 2009). It can be hard, even impossible, to determine the source from which an error has propagated (Buza *et al.*, 2008) causing significant problems for biologists. Annotation quality is not consistent across all databases and annotators (Dolan *et al.*, 2005), whether curated manually or automatically. It can, therefore, be difficult to determine the level of quality, maturity or correctness of a given textual annotation. However users often incorrectly assume that annotations are of consistent quality and correctness (Ussery and Hallin, 2004).

Currently there are few standard metrics for assessing annotation quality. Annotations are frequently assigned a score, using a variety of methods. These approaches include assigning confidence scores to annotations based on their stability (Gross *et al.*, 2009) or combining the breadth (coverage of gene product) and the depth (level of detail) for the terms in the Gene Ontology (GO) (Buza *et al.* 2008). However, while deeper nodes within an ontology are generally more specialized, these measures are problematic; first GO has three root domains and second an ontology, such as GO, is a graph not a tree, therefore depth is not necessarily meaningful. Other methods (Buza *et al.*, 2008; Pal and Eisenberg, 2005; Rogers and Ben-Hur, 2009) use evidence codes as a basis for an annotations reliability, although ironically, the GO annotation manual explicitly states that evidence codes should NOT be used in this way (GO Consortium, 2011), describing rather the type of evidence not its strength.

All of these approaches rely upon additional information to determine annotation quality. Resources, such as sequence databases, vary in their structures and in the additional information stored. For example, not all resources use evidence codes and these codes are not comparable between resources (Lord *et al.*, 2003); likewise, it is not generally possible to use methods based on an ontological hierarchy for non-ontological resources.

Most resources carry some annotations which are unstructured, free text. Therefore, a quality metric that can be derived purely from textual annotation would potentially allow any resource to be analysed and scored. There are various measures for analysing the quality of text, such as the Flesch–Kincaid Readability Test (Flesch, 1948) and SMOG Grading (Laughlin, 1969). These metrics are generally based around readability, or reading-age; that is the literary quality of the text, rather than correctness and quality of the subject matter.

In this article, we report on a bulk analysis of textual annotation in the UniProt Knowledgebase (UniProtKB), attempting to understand whether we can exploit our knowledge of changes in the annotation over time as a mechanism for developing a quality measure of biological correctness. We investigate word occurrences, and their changes over time, as reflected in their distribution; we show that using these relationships we are able to detect large-scale changes in the annotation process; and we demonstrate that the parameters of these relationships also change. Specifically, we fit a power-law distribution to the extracted word occurrences and extract a value, called *α*. We relate this *α* value to Zipf's principle of least effort, which states it is human nature to take the path of least effort to achieve a goal. Broadly, higher values of *α* indicate a resource which is easier for the reader, whereas lower values are easier for the annotator.

Although manually curated annotation is generally accepted as the most accurate (Curwen *et al.*, 2004), a significant problem is the lack of more explicit gold standard datasets (James *et al.*, 2011; Roberts *et al*., 2011). This makes defining a quality measure somewhat troublesome. Our investigation into whether changes in word distribution are representative of quality and maturity in the annotation show that these forms of measures can detect large-scale features of annotation, and that clear trends appear as UniProtKB matures and grows over time. These trends are often reflective of our a priori judgements of quality within UniProtKB, for example, a distinction between manual and automated annotation. In the absence of a gold standard, we believe that this represents reasonable evidence that this form of analysis may be used as the basis for a quality metric for textual annotation.

## 2 METHODS

### 2.1 Data extraction from UniProtKB

UniProtKB (UniProt Consortium, 2010) consists of two sections: UniProtKB/Swiss-Prot, which is reviewed and manually annotated, and UniProtKB/TrEMBL which is unreviewed and automatically annotated. The first version of Swiss-Prot was released in 1986, with TrEMBL appearing in 1996. Releases of TrEMBL were initially more frequent than Swiss-Prot, meaning the databases were released independently. In Table 1, we map between each Swiss-Prot release and the nearest version of TrEMBL. This allows us to compare the quality of manually and automatically curated annotation at similar points in time.

**Table 1.** Mapping between TrEMBL and Swiss-Prot release dates

Date	Swiss-Prot version	Date	TrEMBL version
Oct-96	34	Nov-96	1
Nov-97	35	Jan-98	5
Jul-98	36	Aug-98	7
Dec-98	37	Jan-99	9
Jul-99	38	Aug-99	11
May-00	39	May-00	13
Oct-01	40	Oct-01	18
Feb-03	41	Mar-03	23
Oct-03	42	Oct-03	25
Mar-04	43	Mar-04	26

For each version of Swiss-Prot, we have associated the nearest version of TrEMBL based on release date.

Following the formation of the UniProt Consortium in 2002, the releases of the two databases were synchronized (from 2004). The correct names are now technically UniProtKB/Swiss-Prot and UniProtKB/TrEMBL. We will use the following naming approach for clarity:

- **UniProt**—Refers to the UniProt Consortium.
- **Swiss-Prot**—Refers to Swiss-Prot entries prior to the formation of the UniProt Consortium.
- **TrEMBL**—Refers to TrEMBL entries prior to the formation of the UniProt Consortium.
- **UniProtKB**—Refers to the combination of both Swiss-Prot and TrEMBL datasets.

Where necessary we will explicitly write UniProtKB/Swiss-Prot or UniProtKB/TrEMBL. This naming scheme allows us to refer to post-UniProtKB versions of UniProtKB/Swiss-Prot and UniProtKB/TrEMBL with the same number, starting from version two of UniProtKB.^1^ We can investigate annotation change over time, as complete datasets for historical versions of UniProtKB and Swiss-Prot are made available by UniProt on their FTP server, with the exception of Swiss-Prot versions 1-8 and 10 which were never archived. Pre-UniProtKB/TrEMBL releases were kindly made available to us by UniProt.

**Fig. 1.**
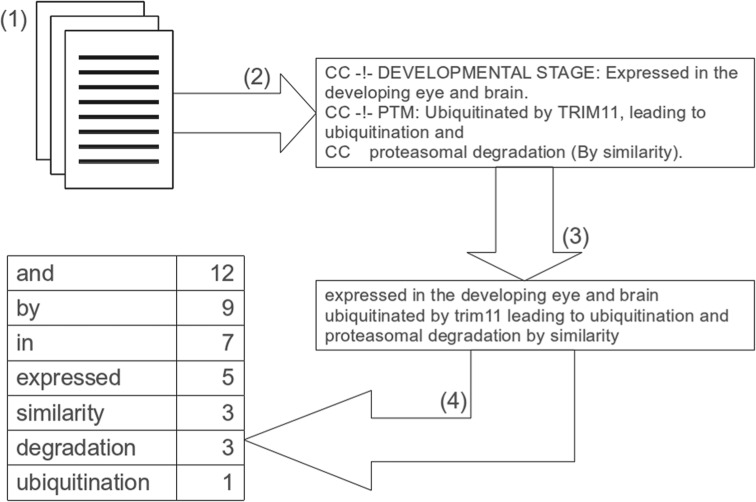
Outline view of the data extraction process. (1) Initially we download a complete dataset for a given database version in flat file format. (2) We then extract the comment lines (lines beginning with ‘CC’, the comment indicator). (3) We remove comment blocks and properties [as defined in the UniProtKB manual (UniProt Consortium, 2011)], punctuation, ‘CC’, brackets and make words lower case, so as to treat them as case insensitive. (4) Finally, we count the individual words and update the occurrence of each word total count

Our extraction approach, also summarized in Figure 1, involves four key steps:

1. The UniProt FTP server (ftp.uniprot.org/pub/databases/uniprot/) provides complete datasets for past versions of Swiss-Prot and UniProtKB in flat file format.
2. UniProtKB flat files adhere to a strict structure, as detailed in the UniProtKB user manual (UniProt Consortium, 2011). A Java framework was created that allowed UniProtKB comment lines to be correctly extracted.
3. Over time annotations in UniProtKB have become more structured with the addition of topic headings (e.g. ‘subcellular location’ and ‘function’). These headings are ignored by our analysis. We also remove punctuation, the ‘CC’ identifier, brackets and whitespace.
4. The final step in this process is to output a list of all words and their frequency for all annotations in a given database version.

In order to ensure accurate data extraction, we checked that the number of entries parsed (Figure 1, step 2) matched the number expected from the release notes. Additionally the list of headings (comment blocks and properties) removed (Figure 1, step 3) were noted, along with their frequency, to ensure only headings described in the UniProtKB manual were removed. Finally, a random selection of records were manually checked against parsed outputs (Figure 1, all steps).

## 2.2 Model fitting

When developing a framework to model the occurrence of words, a variety of competing models were considered. These ranged from relatively simple distributions, such as the exponential and log-normal, to more complex mixture models. However, the power-law distribution achieved a good balance between model parsimony and fit.

In this article, we only deal with the discrete power-law distribution [see (Clauset *et al.*, 2009) for a discussion on power-laws]. The discrete power-law distribution has probability mass function

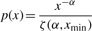

where

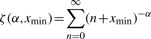

is the generalized or Hurwitz zeta function.

To fit the power-law distribution, we followed the Bayesian paradigm. We assumed a proper uniform *U*(1,5) prior for *α*. Since the posterior distribution for the parameters is analytically intractable, we integrate out the uncertainty using a Markov chain Monte Carlo (MCMC) algorithm. The parameter space was explored using a Gaussian random walk. The Markov chain reached equilibrium very quickly and only a small amount of thinning was necessary.

We modelled multiple datasets simultaneously using a ‘fixed-effects’ approach. Let *i* denote the dataset of interest, then we aim to infer the parameter

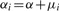

where *α* is the coefficient for a baseline dataset and *μ_i_* is difference from this baseline. For example in Figure 3, the baseline dataset is UniProtKB/Swiss-Prot version 16 and *μ_i_* represents the change in the *α* coefficient from UniProtKB/Swiss-Prot version 16.

Throughout this article, we set *x*_min_ = 50, which we determined using the Bayesian information criterion (BIC) criteria when fitting all the datasets in Figure 3. However, the conclusions are not sensitive to changes of *x*_min_. For smaller values of *x*_min_, the credible region is reduced since there is more data, conversely increasing *x*_min_ to around 200 increases the credible regions slightly.

The fitting of a power-law corresponds to the exponent of the regression line represented by *α*. Given that the graph and *α* value is based on the underlying text, it is plausible that the *α* value could provide a measurement to assess the underlying textual quality. Indeed, it has been previously suggested (Ferrer, 2005) that the *α* value is related to Zipf's principle of least effort (Zipf, 1949).^2^ This principle states that it is human nature to take the path of least effort when achieving a goal. For example, an annotator can create an annotation with generic terms (least effort for the annotator, more work for the reader) or with precise and specialist terms (least effort for the reader, more work for the annotator). Table 2 shows *α* values that have been extracted from a variety of texts, that give confidence to this claim. We can use this information as a basis for quality; texts which require minimal effort for the reader, due to expertly curated annotation, are deemed to be of high quality.

**Table 2.** Relationship between *α* value and Zipf's principle of least effort

*α* value	Examples in literature	Least effort for
*α* < 1.6	Advanced schizophrenia (Piotrowska and Piotrowska, 2004; Zipf, 1949), young children (Brillouin, 2004; Piotrowska and Piotrowska, 2004)	–
1.6≤ *α* < 2	Military combat texts (Piotrowska and Piotrowska, 2004), Wikipedia (Serrano *et al.*, 2009), Web pages listed on the open directory project (Serrano *et al.*, 2009)	Annotator
*α* = 2	Single author texts (Balasubrahmanyan and Naranan, 1996)	Equal effort levels
2< *α* ≤ 2.4	Multi author texts (Ferrericancho, 2005)	Audience
*α* > 2. 4	Fragmented discourse schizophrenia (Piotrowska and Piotrowska, 2004)	–

For *α* values *<* 1.6 or *>* 2.4, we have no corresponding effort–level as the text is treated as incomprehensible.

## 3 RESULTS

### 3.1 Does annotation in UniProtKB obey a power-law distribution?

**Fig. 2.**
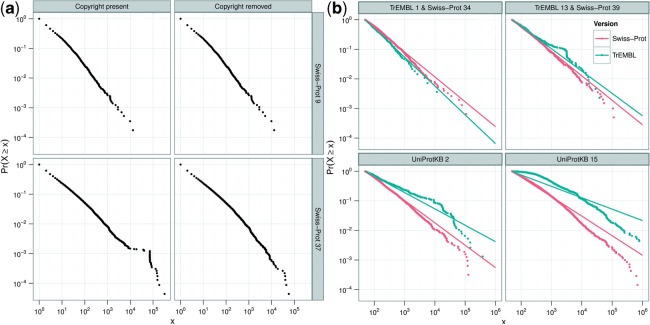
Cumulative distributions of words for various Swiss-Prot and TrEMBL versions, shown with logarithmic scales. The size (number of words) is shown along the *X*-axis whereas the probability is shown on the *Y*-axis. A point on the graph represents the probability that a word will occur *x* or more times. For example, the upper left most point represents the probability of 1 (i.e. 10^0^) that a given word will occur once (i.e. 10^0^) or more times. A word must occur at least once to be included. Words occurring very frequently are presented in the bottom right of the graph. (**a**) Shows the resulting graphs for Swiss-Prot version 9 (November 1988) and Swiss-Prot version 37 (December 1998), with and without copyright. The distinct structure visible between *x* = 10^4^ and *x* = 10^5^ in Swiss-Prot version 37 (bottom left panel) is caused by the copyright statement declaration. Swiss-Prot version 9 operates as a control to show that the attempted removal of copyright has no effect where no copyright information is present. (**b**) Shows the data with fitted power-law distributions for an even subset of historical versions of Swiss-Prot and the co-ordinate release of TrEMBL

Power-laws have been shown to exist in numerous man-made and natural phenomena (Clauset *et al.*, 2009). The link between Zipf's principle of least effort and *α* was originally based on natural language. If a power-law distribution is a measure of quality, we would expect that a power-law distribution is more likely to occur in human-curated annotation rather than annotations produced automatically. Therefore, for our initial analysis, we selected Swiss-Prot as a gold standard resource. Results are shown in Figure 2a, for two versions of Swiss-Prot. We can see that annotation does broadly obey a power-law, although with a distinct ‘kink’ in Swiss-Prot version 37, between *x* = 10^4^ and *x* = 10^5^.

Inspection of the words in this region showed that this structure is artefactual, resulting not from annotation *per se* but from copyright and license information, which are included in the CC lines, along with biological information. These copyright statements were first introduced into Swiss-Prot at version 37, with wording changes at UniProtKB versions 4 and 7. From this analysis, we show that we can detect the introduction of a large amount of material with no biological significance into the annotation. This demonstrates that the power-law can be used as a partial measure of quality, albeit for detecting artefacts.

For subsequent analysis we removed the copyright statements. Updated graphs, with copyright statements removed, are also shown in Figure 2a. Inspection of these graphs show that the slope for the head and tail increase at different rates with Swiss-Prot versions. This is a result of a marked two-slope behaviour which is commonly seen for mature resources, such as large complex natural languages (Cancho and Solé, 2001; Ha *et al.*, 2006). These graphs follow a power-law distribution reasonably well. However Figure 2b shows some versions of TrEMBL where a power-law does not fit that well. As discussed in Section 2.2, various competing models were considered, with a power-law being chosen partly due to the simplicity of its output, *α*. It is clear that this change over time requires further analysis.

### 3.2 How do the distributions change over time?

**Fig. 3.**
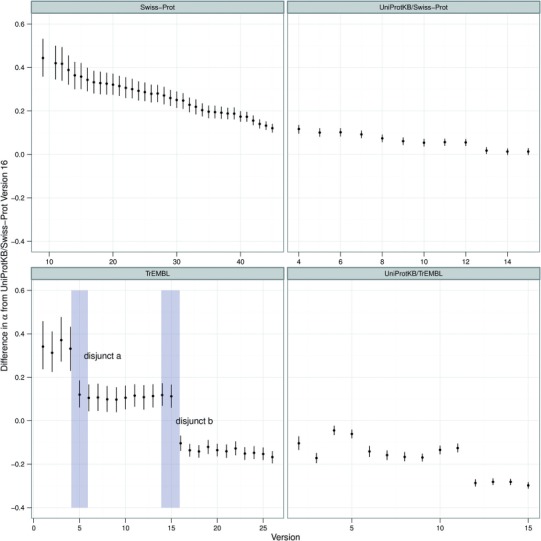
*α* values over time, for each version of Swiss-Prot and TrEMBL. The graph shows the difference in *α* value (with 95% credible region) from UniProtKB/Swiss-Prot version 16, for which the *α* value was 1.62. So, for example, Swiss-Prot version 9 has a difference of, approximately, 0.45. Therefore the resulting *α* for Swiss-Prot version 9 is around 2.07

Although it is useful for demonstrating the two-slope behaviour, this view makes it difficult to see change over time. Given that the main analytical value comes from the extracted *α* values, subsequent graphs show just these values for different database versions. This approach allows us to investigate the change over time by looking at all historical data simultaneously. The resulting graphs from this analysis is shown in the top half of Figure 3.

As Figure 3 shows, the annotation in Swiss-Prot is changing in its nature over time. *α* decreases over time for Swiss-Prot, suggesting that Swiss-Prot is becoming optimized towards least effort for the annotator, rather than the reader. This fits with previous research from Baumgartner *et al.* (2007) suggesting that the enormous increase in proteins requiring annotation is outstripping the provision of this annotation. This issue has been acknowledged by UniProt, with their introduction of automated annotation; therefore, we next investigate this form of annotation.

### 3.3 How does manual annotation compare to automated annotation?

Within UniProtKB proteins are initially annotated automatically and placed into TrEMBL. Eventually they are manually annotated and placed into Swiss-Prot. Therefore, TrEMBL and Swiss-Prot are ideal resources to compare equivalent human and automated annotations. Here, we compare these two resources, investigating their behaviour over time, at equivalent points in time. As previously described, prior to UniProtKB version two, TrEMBL and Swiss-Prot releases were not synchronized, so we use the version of TrEMBL released closest in time to each version of Swiss-Prot, as show in Table 1. An evenly spaced subset of these analyses are shown in Figure 2b, with Figure 3 showing the *α* values for all versions of TrEMBL and Swiss-Prot.

In Figure 2b, TrEMBL and Swiss-Prot appear to diverge over time with Swiss-Prot demonstrating the behaviour typical of a more mature resource. TrEMBL shows less maturity, with many words occurring with a high frequency. In short, Swiss-Prot appears to show a richer use of vocabulary. We cannot, however, rule out the possibility that this difference occurs as they are annotating different proteins. Unfortunately, it is not possible to check a proteins annotation in both Swiss-Prot and TrEMBL at the same point in time; once a record is migrated to Swiss-Prot, it is removed from subsequent versions of TrEMBL. This is necessary as Swiss-Prot is used as a basis for annotation in TrEMBL, so proteins not removed from TrEMBL would have their automated annotation based on their manual annotation in Swiss-Prot. However, the rapid increase in size of both resources, argues against this explanation.

Whereas Swiss-Prot shows a relatively regular progression, TrEMBL does not. There are two significant disjuncts in the relationship where large jumps occur between releases, as highlighted in Figure 3. We also note a significant rise of total words between these versions, compared to those for nearby releases (data not shown).

We have identified two plausible explanations for these disjuncts, based on historical events. Firstly in 1998 (highlighted by *disjunct a*) a number of new procedures appear to have been introduced (Bairoch and Apweiler, 1998). These approaches include making use of the ENZYME database, specialized genomic databases and scanning for PROSITE patterns compatible with an entries taxonomic range. PROSITE patterns are used to enhance the content of the comment lines by adding information such as protein function and subcellular location. Interestingly, prior to this disjunct, the first four versions of TrEMBL have an *α* value higher than their Swiss-Prot counterpart.

Secondly in 2000, the introduction and development of annotation rules was planned in TrEMBL which could explain the second jump (highlighted by *disjunct b*) (Bairoch and Apweiler, 2000). Both of these disjuncts would be expected to produce an increase in the total amount of annotation, as well as introducing new words and phrases which would affect the measures described here. Given the lack of detailed statistics and the age of the database at this time, UniProt could not confirm these explanations. They did acknowledge that extensive work in 2001 and early 2002 was carried out to improve the data, although they believe the scanning of PROSITE was in effect from TrEMBL version 1.

The increase of total words noted earlier correlates with the increase of entries into UniProtKB; the rate of data being added is exponential. Given this increase, we find ourselves analysing entries and annotations of mixed age. Here we have seen the apparent decreasing of quality for complete datasets, of both Swiss-Prot and TrEMBL, over time. Following on from this, we wish to explore the quality of annotations within a set of mature entries.

### 3.4 Analysing maturity of entries over time and the impact of new annotations

**Fig. 4.**
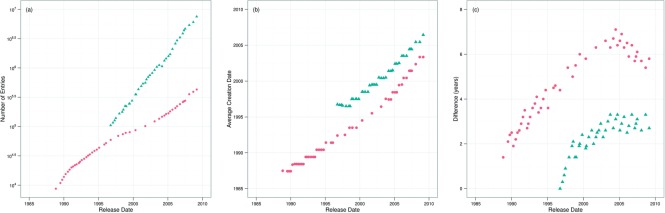
Swiss-Prot (red circles) and TrEMBL (blue triangles). (**a**) Growth (number of entries) in Swiss-Prot and TrEMBL over time. (**b**) Average creation date over time for Swiss-Prot and TrEMBL. (**c**) Difference between release date and average creation date (i.e. age) over time

Our prior analysis has investigated annotation quality in bulk, without analysing how individual records are maturing. If we consider ‘maturity’ as a simple function of age, then we would expect, given the rapid increase in the size of Swiss-Prot, while new records appear, the older entries should mature. Figure 4a illustrates the exponential rate at which Swiss-Prot and TrEMBL are growing, showing the number of entries in each database version.

Each entry contains a date stamp, indicating when it was first introduced into the database. We use this information to show that the average creation date of a record has increased only slowly over the life span of Swiss-Prot as a whole—illustrated in Figure 4b. Swiss-Prot is currently around 20 years old, yet the average record age is around 5 years old and decreasing. This is illustrated in Figure 4c, where we show the difference between the average creation date and release date. As an example, Swiss-Prot version 9 was released in November 1988 and the average entry release date is July 1987, so the figure reflects this difference of 1 year and 4 months. Given this, we wished to abstract from increasing size of Swiss-Prot and ask whether individual records appear to be maturing.

**Fig. 5.**
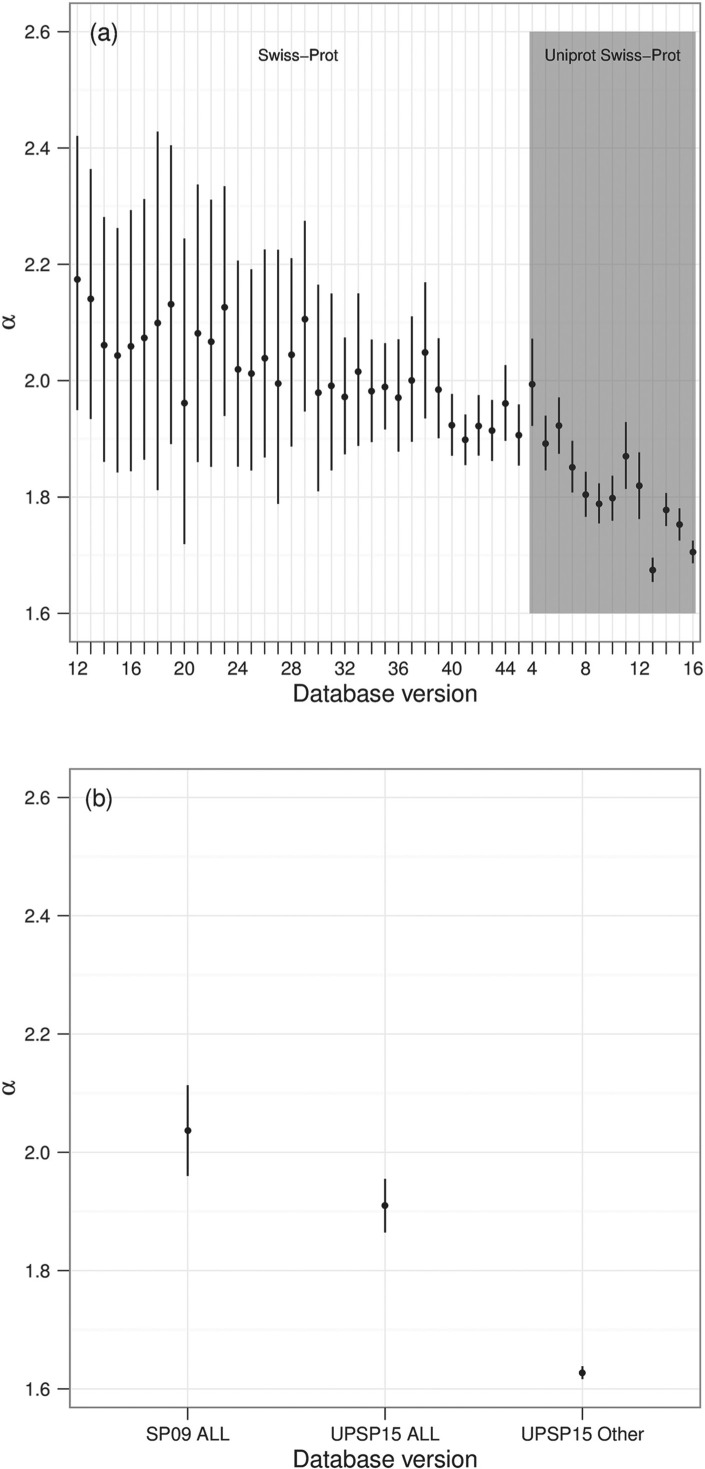
(**a**) Analysis of those entries that are new to a particular version of Swiss-Prot. (**b**) *α* value (with 95% credible region) for all entries in Swiss-Prot version 9 that are in UniProtKB version 15, all entries in UniProtKB version 15 that are in Swiss-Prot version 9, and all those in UniProtKB version 15, but not in Swiss-Prot version 9

This analysis is not straightforward; we need, essentially, a set of records which relate to a defined set of proteins. To achieve this we extracted the annotations from all of the entries that were common in both Swiss-Prot version 9 and UniProtKB/Swiss-Prot version 15 (the first and last version available to us). The resulting *α* values are shown in Figure 5b, with the addition of the *α* value for those entries in UniProtKB version 15 but not Swiss-Prot version 9. These results show that the *α* value for the mature set of entries has decreased over time, correlating with the Swiss-Prot database as a whole.

Given that the *α* value for mature entries has decreased over time, it is of interest to investigate the *α* values of entries that are new to each version of Swiss-Prot. For this, we extracted annotations from entries that appeared for the first time in a given database version. Results of this analysis are shown in Figure 5a. It again would appear that the *α* value is decreasing over time, similar to that of other Swiss-Prot graphs.

## 4 DISCUSSION

The biological community lacks a generic quality metric that allows biological annotation to be quantitatively assessed and compared. In this article we applied power-law distributions to the UniProtKB database and linked the extracted *α* values to Zipf's principle of least effort in an attempt to derive such a generic quality metric. The results within this article give confidence to our initial hypothesis that this approach holds promise as a quality metric for textual annotation.

Initially, our analysis focused on the manually curated Swiss-Prot. As shown in Figure 3, early versions of Swiss-Prot give *α* values that suggest annotations were of high quality; that is, they were of least effort for the reader. However, over time we see a steady reduction in the *α* value, which does suggest that the average annotation is now harder for readers to interpret, requiring more expertize to consume the data than was previously required. This result is perhaps best explained by the exponential increase of data that is added to Swiss-Prot. Manual annotations are regarded as the highest quality annotation available and it is inevitable that the acknowledged pressure on manual annotation is going to increase. This conclusion appears to fit with previous research (Baumgartner *et al.*, 2007) that shows manual curation techniques cannot keep up with the increasing rate of data.

Many of the patterns exhibited by Swiss-Prot are also shown in our analysis of TrEMBL. From Figure 3, we conclude that annotation in Swiss-Prot and TrEMBL show similar characteristics in that, for both cases, annotation appears to be increasingly optimized to minimize efforts for the annotator rather than the reader; unsurprisingly, this appears to be more pronounced for TrEMBL than for Swiss-Prot. We are currently unclear whether this form of direct numerical comparison over the two different resources is highly meaningful, although this distinction between the two resources appears to be more pronounced over time rather than less. Therefore, these results are consistent with the conclusion that manual annotation behaves as a significantly more mature language than automated annotation. This fits with our a priori assumptions which again is suggestive that this form of analysis is operating as a measure of quality.

In addition to analysing whole UniProtKB datasets, we also investigated how sets of entries mature over time (Fig. 5b) and the quality of annotations within new entries (Fig. 5a). Within the mature entries we interestingly see a decrease in quality over time, rather than increasing or maintaining a similar quality level. However this decrease is much slower than the Swiss-Prot database as a whole over time, and is still of much higher quality than the remainder of entries in UniProtKB version 15. For the new annotations we also see a general decrease in quality over time. It is plausible that these results stem from the manner of management and curation of annotations; the UniProt annotation protocol consists of six key steps (Magrane and Uniprot Consortium, 2011), one of which is identifying similar entries (from the same gene and homologues by using BLAST against UniProtKB, UniRefs and phylogenomic resources). If two entries from the same gene and species are identified then they are merged; annotations between the remaining entries are then standardized. It would appear that attempts to standardize growing sets of similar entries is having a detrimental effect on the quality of both individual entries and the overall database.

In addition to being used as a quality measure, the approach described here could be used for artefactual error detection. Our early analysis identified information with no biological significance (copyright statements) included within the comment lines.

Our focus in this article was on UniProtKB, and we have not tested across other databases. One database of immediate interest would be InterPro. The work in this article focuses on protein annotation; extending this to InterPro would allow us to analyse protein family annotation which would normalize for the many near duplicate records of the large protein families found in UniProtKB. This would require further bulk analysis—however, once data are correctly extracted, this form of analysis is straightforward and does not require specialist resources. Analysis of other forms of annotation would also be interesting; Kalankesh *et al.* (2012) has recently reported on similar results in GO annotation.

Further work analysing additional databases would allow us to draw more conclusive conclusions regarding the fitting of the power-law, and consequently the usability of *α* as a quality metric. However, our analysis of UniProtKB suggests that this approach holds promise of being a useful tool for database curators and users alike.

## Footnotes

^1^Version 2 was the first major release containing Swiss-Prot version 44 and TrEMBL version 27.

^2^Additionally, Zipf has shown that a words occurrence is inversely proportional to its rank (Zipf's Law). Zipf's law, Pareto's law and power-law distributions are all types of power-law that are, essentially, different ways of looking at the same thing (Adamic and Huberman, 2002).
